# The effect of whole-body vibration exercise on postmenopausal women with osteoporosis: A protocol for systematic review and meta-analysis

**DOI:** 10.1097/MD.0000000000025606

**Published:** 2021-05-07

**Authors:** Shengqin Cao, Zhongfang Wang, Chongyang Li, Qiaoli Wang

**Affiliations:** a The Fourth People's Hospital of Jinan City, Jinan; b Binzhou Medical University, Binzhou, Shandong Province, China.

**Keywords:** osteoporosis, postmenopausal, protocol, systematic review, whole-body vibration

## Abstract

**Background:**

Osteoporosis (OP) is an age-related disease characterized by reduced bone mass and increased bone fragility. It is more common in older people and postmenopausal women. As a new type of exercise training for OP, whole-body vibration (WBV) exercise has been proved to have a good effect on postmenopausal women with OP. It can increase bone density and improve strength and balance in postmenopausal population, which has certain clinical value, but lacks evidence-based medicine evidence. This study aims to systematically study the effectiveness of WBV exercise on postmenopausal women with OP.

**Methods:**

The English databases (PubMed, Embase, Web of Science, The Cochrane Library) and Chinese databases (China National Knowledge Network, Wanfang, Weipu, China Biomedical Database) were searched by computer. From the establishment of the database to February 2021, the randomized controlled clinical studies on WBV exercise on postmenopausal women with OP were conducted. The quality of the included studies was independently extracted by 2 researchers and literature quality was evaluated. Meta-analysis of the included studies was performed using RevMan5.3 software.

**Results:**

In this study, the efficacy and safety of WBV exercise on postmenopausal women with OP were evaluated by lumbar spine bone density, femoral neck bone density, pain, incidence of falls, incidence of fractures, and quality of life scale score, etc.

**Conclusion:**

This study will provide reliable evidences for the clinical application of WBV exercise on postmenopausal women with OP.

**Ethics and dissemination:**

Private information from individuals will not be published. This systematic review also does not involve endangering participant rights. Ethical approval will not be required. The results may be published in a peer-reviewed journal or disseminated at relevant conferences.

**OSF Registration number:**

DOI 10.17605/OSF.IO/WPYT9

## 1 Introduction

Osteoporosis (OP) is a generalized bone disease characterized by decreased bone mass and deterioration of bone microarchitecture predisposing to fragility fractures.^[1]^ About 50% of women and 20% of men fracture after age 50 due to low bone density.^[2]^ Postmenopausal osteoporosis (PMOP) is a common type, mainly due to stop of endocrine ovarian function of postmenopausal women and estrogen deficiency, which aggravate the process of bone demineralization, resulting in abnormal bone microstructure, resulting in bone fragility, increase the incidence of fracture.^[3]^ Drug treatment for PMOP mainly includes calcium, vitamin D, selective estrogen receptor modulators, hormone replacement therapy, bisphosphonates, etc^[4]^ to inhibit bone absorption or promote bone formation, but there are drawbacks such as long treatment cycle, many adverse drug reactions, and high treatment cost. However, physical therapy is favored due to its efficacy and safety.^[5]^ Among them, the whole-body vibration (WBV) training, also known as the WBV exercise, as a new type of exercise training for OP, can improve the bone mineral density, exercise capacity, related parameters of patients, improve the strength and balance of postmenopausal people, with the prevention and treatment of PMOP.^[6–8]^

At present, many studies^[9–11]^ have shown that WBV exercise has a positive effect on bone metabolism in postmenopausal women, which can improve muscle and bone parameters, and plays an important role in the prevention and treatment of PMOP. It is worth popularizing in clinical practice. However, there are differences in the research scheme and efficacy of each clinical trial, such as the frequency and amplitude of vibration, the course of disease and the course of treatment, which lead to uneven research results and affect the promotion of this prevention and treatment method to a certain extent. This study aims to investigate the effect of WBV exercise on postmenopausal women with OP. The results of this study will help confirm the effect of WBV exercise on bone density, muscle health, balance ability and functional results of postmenopausal women with OP, and provide reliable reference for clinical application of WBV exercise on postmenopausal women with OP.

## 2 Methods

### 2.1 Protocol register

The protocol for this system review and meta-analysis was drafted under the guidance of the preferred reporting items for systematic reviews and meta-analyses protocols. In addition, it has been registered with the Open Science Framework (OSF) on March 4, 2021. (Registration number: DOI 10.17605/OSF.IO/WPYT9).

### 2.2 Ethics

We did not recruit and collect patient personal information for this protocol and therefore did not require ethics committee's approval.

### 2.3 Eligibility criteria

#### 2.3.1 Types of studies

We collected all available randomized controlled trails on WBV exercise on postmenopausal women with OP. Whether to use blind method; the language was limited to Chinese and English; publication status was unlimited.

#### 2.3.2 Research object

1. Natural menopausal women diagnosed with reduced bone mass or OP by detecting bone mineral density levels in the lumbar spine or femoral neck. Diagnostic criteria: −2.5SD <T value <−1.0SD was hypobone mass; T value ≤2.5SD was OP^[12]^;
2. Secondary OP was excluded;
3. Patients with good physique and no obstacle of motor function;
4. The nationality, race, age, and menopausal age of the patients were not limited.

#### 2.3.3 Interventions

The control group was treated with calcium, vitamins, and other essential anti-osteoporosis drugs, with no specific treatment methods. The observation group was treated with WBV exercise (unlimited frequency, amplitude, vibration equipment, vibration mode, duration, and frequency of treatment) on the basis of treatment in the control group.

#### 2.3.4 Outcome indicators

Main outcome indicators: Lumbar vertebral bone density; Femoral neck bone density. (evaluated by dual-energy X-ray absorption measurement)

Secondary outcome indicators: Pain; Incidence of falls; Incidence of fracture; Serum bone metabolic markers; Whole body fat content; Quality of life scale score, etc.

### 2.4 Exclusion criteria

1. Repeated papers;
2. Articles that published as abstracts or with incomplete data and cannot obtain complete data after contacting the authors;
3. Literatures without related outcome indicators;
4. Patients have high frequency and high intensity physical activity or exercise.

### 2.5 Retrieval strategy

Using “quan shen zhen dong” (WBV), “zhen dong liao fa” (vibration therapy), “gu zhi shu song” (OP), “jue jing hou gu zhi shu song” (PMOP) as Chinese search terms, the search was conducted in Chinese databases, including China National Knowledge Network, Wanfang Data Knowledge Service Platform, Weipu Information Chinese Journal Service Platform, China Biomedical Database; using “whole-body vibration,” “vibration,” “postmenopausal,” “osteoporosis,” “postmenopausal osteoporosis,” “PMOP,” etc as English search terms, the search was conducted in English databases, including PubMed, EMBASE, Web of Science, the Cochrane Library, in addition, manual retrieval was carried out on Baidu Academic and Google Academic. The retrieval time was from the establishment of the database to February 2021. All domestic and foreign literatures on WBV exercise on postmenopausal women with OP were collected. Taking PubMed as an example, the retrieval strategy is shown in Table 1.

**Table 1:** PubMed database retrieval strategy.

Number	Search terms
#1	Osteoporosis [MeSH]
#2	Postmenopausal osteoporosis [MeSH]
#3	Postmenopause osteoporosis [Title/Abstract]
#4	PMOP [MeSH]
#5	#1 OR #2 OR #3 OR #4
#6	Vibration [MeSH]
#7	Whole body vibration [Title/Abstract]
#8	WBV [Title/Abstract]
#9	Vibrotherapy [Title/Abstract]
#10	#6 OR #7 OR #8 OR #9
#11	#5 AND #10

### 2.6 Data screening and extraction

Referring to the research selection method in Cochrane Collaboration Network System Evaluation Manual Version 5.0, according to the PRISMA flow chart, the 2 researchers independently screened and checked the literature using EndNote X9 literature management software according to the above inclusion and exclusion criteria, and checked each other. If there were different opinions, they would negotiate with the third party to resolve differences. Using Excel 2013 to extract relevant information, including:

1. Clinical studies (title, first author, year of publication, sample size, mean age, mean age of menopause, mean course of disease);
2. Intervention measures (type, dose, frequency, and course of anti-osteoporosis drugs in the control group and the observation group; type, frequency, amplitude, duration, and frequency of treatment of WBV in observation group);
3. Risk bias assessment factors in randomized controlled trials;
4. Outcome indicators.

**Figure 1:**
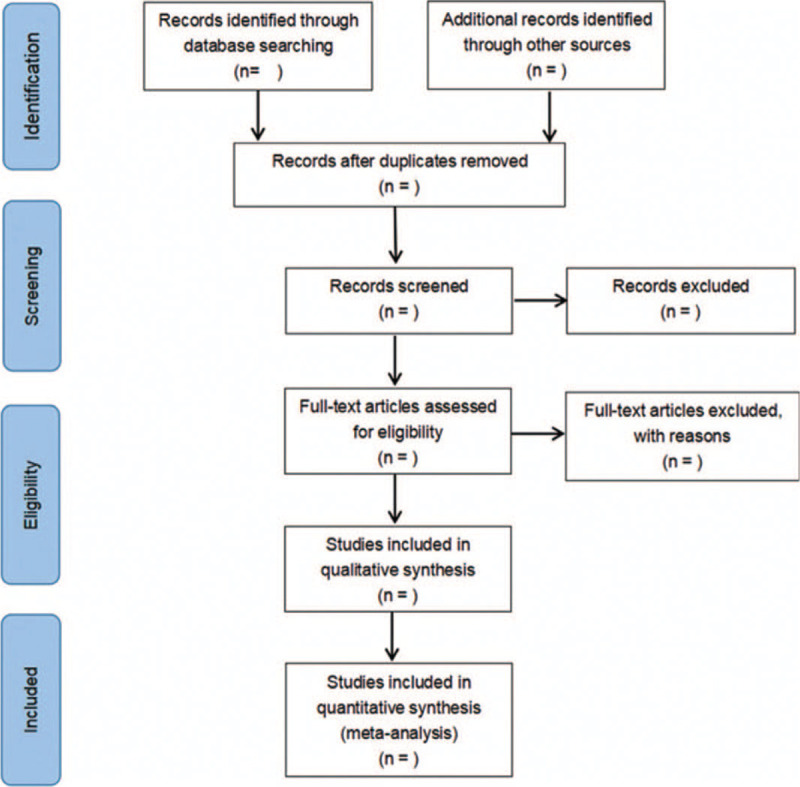
Flow diagram.

The literature screening process is shown in Figure 1.

### 2.7 Literature quality assessment

The risk of bias was assessed using the tools of the Cochrane collaboration to conduct biased risk assessment of included studies. According to the performance of the literature contained in the above evaluation projects, the 2 researchers would give low-risk, unclear or high-risk judgments, and conduct cross-checks after completion. In case of any disagreement, discussion will be carried out. If there were any differences, the discussion would be conducted. If the 2 sides cannot reach agreement, they would discuss with the third party researchers.

### 2.8 Statistical analysis

#### 2.8.1 Data analysis and processing

RevMan5.3 software provided by Cochrane collaboration would be used for statistical analysis. We assumed that the results of different studies depend on the sample variables, covariates and other factors. Fixed effect analysis allowed only inferences similar to those contained in meta-analysis, and random effect analysis allowed more extensive inferences. The overall rate was estimated to be 95% confidence interval and the effect sizes on the meta-regression model. When the interval contained a value of 1 and the *P* value of the effect was greater than 5%, we considered that it has no effect and use the *I*^*2*^ index to measure the heterogeneity of different research results. The index could change between negative values assuming 0% to 100%. The results showed that up to 25% was a small degree of heterogeneity, up to 50% was a medium degree, and 75% and above was a high degree.

#### 2.8.2 Dealing with missing data

If there was no data in this article, we would get more information to the author by email. If the author cannot be contacted, or if the author has lost the relevant data, descriptive analysis, rather than meta-analysis would be conducted.

#### 2.8.3 Subgroup analysis

Subgroup analysis was carried out according to the vibration type, frequency, and amplitude of the WBV exercise; subgroup analysis was carried out according to the type of anti-osteoporosis drugs; subgroup analysis was carried out according to the course of disease.

#### 2.8.4 Sensitivity analysis

Since we noticed the significant heterogeneity of data, we conducted sensitivity analysis by gradually excluding each research.

#### 2.8.5 Assessment of reporting biases

The funnel plot was used to evaluate and analyze publication bias. In addition, Egger and Begg tests were used to assess potential publication bias.

#### 2.8.6 Evidence quality evaluation

We chose to assess the quality of evidence by using the Grading of Recommendations Assessment, Development, and Evaluation, which contains 5 domains, including bias risk, consistency, directness, precision, and publication bias. We divided the evidence into 4 levels: High, Moderate, Low, and Very Low, then adjusted the levels according to the characteristics of the study. We first assumed that RCT studies had a high level of evidence and observational studies had a low level of evidence. Then, we looked for factors that reduced the quality of RCT studies and factors that increased the quality of evidence in observational studies, and finally made a comprehensive judgment.

## 3 Discussion

The 2 major groups of OP are the elderly and postmenopausal women, and menopause and reduced ovarian synthesis of hormones lead to an increased prevalence of OP in postmenopausal women. At present, some scholars believe that physical exercise is considered as a better prevention and rehabilitation method.^[13,14]^ However, for elderly menopausal women, physical exercise may increase the risk of injury. Therefore, vibration therapy composed of low amplitude and high intensity stimulation is a good method, which can safely transmit relevant mechanical signals to patients who cannot exercise to enhance musculoskeletal strength.^[15]^ This mechanical vibration is the catalytic factor of bone formation and bone plasticization. From the perspective of mechanical biology, it affects the growth and differentiation of bone cells with the mechanical stimulation generated by exercise, and improves the health status of bone and joint.^[16]^

Vibration therapy can improve physical properties and reduce the negative effects of aging on bones, muscles and tendons. Low amplitude high frequency vibration is beneficial to muscle strength, posture control, balance ability, new bone formation, spinal bone mineral density, and blood circulation.^[17]^ Vibratory exercise improves bone health by directly triggering bone reaction mechanisms and triggering powerful muscles that strengthen exoskeleton contractions.^[18,19]^ Evidence shows that a small signal is a contributing bone signal when it acts on the bone at a higher frequency.^[20]^ According to the piezoelectric theory, pressure induces bone formation in the potential difference, which acts as an activator of bone formation.^[3]^ Some studies have also shown that WBV exercise can improve the bone strength and bone mineral density of ovariectomized rats, and its mechanism may be achieved by reducing the level of 5-HT in blood.^[21]^

It has been clinically confirmed that WBV exercise on postmenopausal women with OP has certain curative effect. However, the evidence from the randomized controlled trails is inconsistent and the results vary from study to study. For example, most of the research results support the efficacy of WBV exercise in enhancing bone mineral density, while some studies have shown that WBV exercise can improve the maximum extensor knee strength in postmenopausal women with OP, but it has no overall therapeutic effect on bone mineral density, bone conversion indexes, human body measurement parameters or the maximum extensor knee strength.^[22]^ With the increasing number of clinical trials, it is necessary to conduct systematic evaluation on WBV exercise on postmenopausal women with OP. In this study, we will summarize the latest evidence of WBV exercise on postmenopausal women with OP. This work also provides useful evidence for determining whether WBV exercise on postmenopausal women with OP is effective and safe, which is beneficial to clinical practice and health-related decision makers.

However, this systematic review has some limitations. Among the included studies, patients were different in age of menopause, course of disease, degree of OP, application type, frequency, amplitude of vibration therapy, and basic therapy drugs, which may have certain clinical heterogeneity. In addition, the patients’ daily physical activity was not assessed, and the patients’ underlying diseases such as heart disease and diabetes were not taken into account, which may have affected the results.

## Author contributions

**Conceptualization:** Shengqin Cao, Qiaoli Wang.

**Data curation:** Shengqin Cao, Zhongfang Wang, Chongyang Li.

**Formal analysis:** Chongyang Li.

**Funding acquisition:** Shengqin Cao, Qiaoli Wang.

**Investigation:** Chongyang Li.

**Project administration:** Shengqin Cao, Qiaoli Wang.

**Resources:** Shengqin Cao and Qiaoli Wang

**Software operating:** Chongyang Li

**Supervision:** Shengqin Cao, Zhongfang Wang, and Chongyang Li.

**Validation:** Zhongfang Wang, Chongyang Li.

**Visualization:** Zhongfang Wang, Chongyang Li.

**Writing – original draft:** Shengqin Cao, Qiaoli Wang.

**Writing – review & editing:** Shengqin Cao, Qiaoli Wang.
